# Predictors of Outcome After Endovascular Thrombectomy in Acute Basilar Artery Occlusion and the 6hr Time Window to Recanalization

**DOI:** 10.3389/fneur.2019.00923

**Published:** 2019-09-25

**Authors:** Johannes Ravindren, Marta Aguilar Pérez, Victoria Hellstern, Pervinder Bhogal, Hansjörg Bäzner, Hans Henkes

**Affiliations:** ^1^Neuroradiological Clinic, Neurocenter, Klinikum Stuttgart, Stuttgart, Germany; ^2^Neurological Clinic, Neurocenter, Klinikum Stuttgart, Stuttgart, Germany; ^3^Department of Neuroradiology, St Bartholomew's Hospital, London, United Kingdom; ^4^Medical Faculty, University Duisburg-Essen, Essen, Germany

**Keywords:** stroke, posterior circulation, outcome, basilar artery occlusion, time window to treatment

## Abstract

**Background and Purpose:** Decision algorithms for large vessel occlusions in the anterior circulation remain unconfirmed for acute basilar artery occlusion (aBAO). The aim of this study was to investigate procedural parameters, patient characteristics, functional outcome, and survival in dependency of the time window to recanalization from symptom onset. Furthermore predictors of outcome were identified.

**Materials and Methods:** Retrospectively 231 patients with aBAO treated with endovascular treatment (EVT) between November 2008 and February 2019 were identified in a prospectively maintained single center stroke database. Baseline characteristics such as age, cardiovascular risk factors, NIHSS at admission, pre-interventional PC-ASPECTS, periprocedural parameters such as time to recanalization, duration of treatment, extent of reperfusion, collateral status, and occlusion patterns, as well as post-interventional 24 h NIHSS and post-interventional ICH were evaluated. Target variables were mRS at 90 days and mortality over 90 days.

**Results:** Good outcome (mRS 0–2) was attained in 29.5% (*n* = 68) of patients, overall mortality was 36.8% (*n* = 85). In mulitivariate analyses patients with time to reperfusion beyond 6 h had a more than half fold decreased chance of good outcome [OR 0.47 95% CI (0.23–0.95) *p* < 0.05]. The odds for good outcome were reduced by almost 2/3 if post-interventional imaging revealed intracerebral hemorrhage [OR 0.28 95% CI (0.08–0.98)]. Unfavorable outcome was noted in 100% (*n* = 14) of patients with symptomatic ICH. Risk for death was reduced by more than 80% if collaterals were present [0.16 95% CI (0.03–0.87)] and if recanalization was successful (TICI 2b-3) [OR 0.19 95% CI (0.05–0.78)]. The odds for survival were 5-fold higher in patients with no post-interventional hemorrhages present [OR 5.35 95% CI (2.2–1.58)].

**Conclusion:** This study might contribute to explaining the ambiguous findings regarding the validity of the 6 h time window in aBAO, suggesting that collateral status impacts the odds of survival in the time window to recanalization beyond 6 h. In our study recanalization within 6 h from symptom onset was associated with good outcome. Successful recanalization (TICI 2b-3a) was necessary for good outcome and survival, post-interventional ICH was highly associated with unfavorable outcome. This might ease the decision making for EVT.

## Introduction

The overwhelmingly positive trial results favoring mechanical thrombectomy over thrombolysis have represented one of the most important advances in stroke care for decades. However, there are still numerous questions that need to be answered. The positive trials all enrolled patients with anterior circulation strokes and therefore, the advantage of mechanical thrombectomy in large vessel thromboembolic occlusion in the posterior circulation is yet to be determined. Acute basilar artery occlusion (aBAO) is one of the most devastating subtypes of stroke with a mortality rate of more than 30% following mechanical thrombectomy (mTE) (1, 2). The factors relating to this high level of mortality are still unknown. However, this should be compared to mortality rates of up to 90% if left untreated (3, 4) as well as high rates of ambulatory outcome (3, 5, 6). As aBAO accounts for only 1% of all stroke types (7) both data collection and designing randomized controlled trials are challenging. Appropriate selection of patients for mTE remains problematic using the data currently available. Nevertheless, despite methodological limitations, successful recanalization in aBAO is endorsed as a key factor for survival and functional outcome (8–10) as recently reported in a study with 51 patients, IV thrombolysis alone failed to achieve recanalization in thrombi exceeding a length of 13 mm in aBAO (11). This study is based on data from a large real-world single center registry with 231 patients who underwent endovascular treatment (EVT). The purpose was to investigate the ambiguous findings regarding the time window from symptom onset to treatment. Therefore, procedural parameters and patient characteristics including collateral status and pathophysiological properties such as occlusion patterns were assessed and stratified by time to recanalization\reperfusion (TICI 2b-3) (TTR) from symptom onset. Functional outcome and survival after 90 days were studied, stratified by the time window to treatment.

## Methods

We retrospectively identified all consecutive patients in our prospectively maintained single center stroke database who had undergone EVT for an aBAO between November 2008 and February 2019. Inclusion criteria included proven aBAO on CT or MR angiography, treatment with or without IV rtPA, use of accepted endovascular treatment strategies with either stent-retrievers, aspiration or a combination of both. No limit was placed on the admission NIHSS or the age of the patient. Exclusion criteria were if patients had a basilar artery occlusion due to an aneurysm, dissection, in-stent thrombosis, non-occlusive thrombi, and failed attempts as well as manifest brain tissue damage not compatible with life or spontaneous recanalization in aBAO. In total 46 patients were excluded. Endovascular treatment is always our first line treatment for an aBAO and was attempted regardless of age, time from stroke onset and unknown stroke onset. Patients had either been admitted directly to our comprehensive tertiary care neurological center or had arrived as inter-hospital transfers from secondary care hospitals in surrounding areas after undergoing baseline imaging (computed tomography or magnetic resonance imaging). All patients underwent an mTE using either stent-retrievers or aspiration.

### Study Population

*Baseline* characteristics including demographics (age and sex), cardiovascular risk factors including smoking, cholesterol levels, hypertension, diabetes mellitus, and the NIHSS at admission were recorded. Furthermore, an Alberta Stroke Program Early CT Score (PC-ASPECTS) for the posterior circulation was derived from each patient's CT or MRI scan by an independent reviewer (JR) not party to any of our patient information, according to the method described in literature (12).

The time of symptom onset to recanalization (TTR) was recorded and categorized into up to 6 h and over 6 h, with the latter group including patients with unknown time of onset and last seen well beyond 6 h.

### Endovascular Procedures

*All patients were treated in* the neuro-angiography suites under general anesthesia and were therefore sedated and intubated irrespective of their clinical status at admission. The devices used to achieve recanalization were at the discretion of the operator. Procedures were performed via the right common femoral route as standard using a 6F sheath. A brachial access route was used in patients if femoral access was not successful. Balloon guide catheters were not used as standard due to the location of the occlusions in the posterior circulation. The standard guide catheters included 6F Guider Softip (Boston Scientific) and 5F Envoy (Cordis). The microcatheters used in mechanical thrombectomy were either RapidTransit or Prowler Select Plus (Cordis, now Cerenovus); Trevo pro 18 MC (Stryker) or Velocity (Penumbra), The standard microguidewires were Synchro2 0.014” (Stryker) or pORTAL (phenox). Navien A+ 0.058” ID (Medtronic) and SOFIA Plus 0.070” ID (Microvention) were used for aspiration thrombectomies. Mechanical thrombectomy was performed with Solitaire FR (Medtronic) or pRESET (phenox). The earliest cases in the subset (2008–2009) used pCRC (phenox) or BONnet (phenox)devices.

The procedure time was defined as groin puncture time to final angiographic run. The date of treatment was recorded to test for any longitudinal effect on the clinical outcome.

### Anatomical Assessment

**Figure 1:**
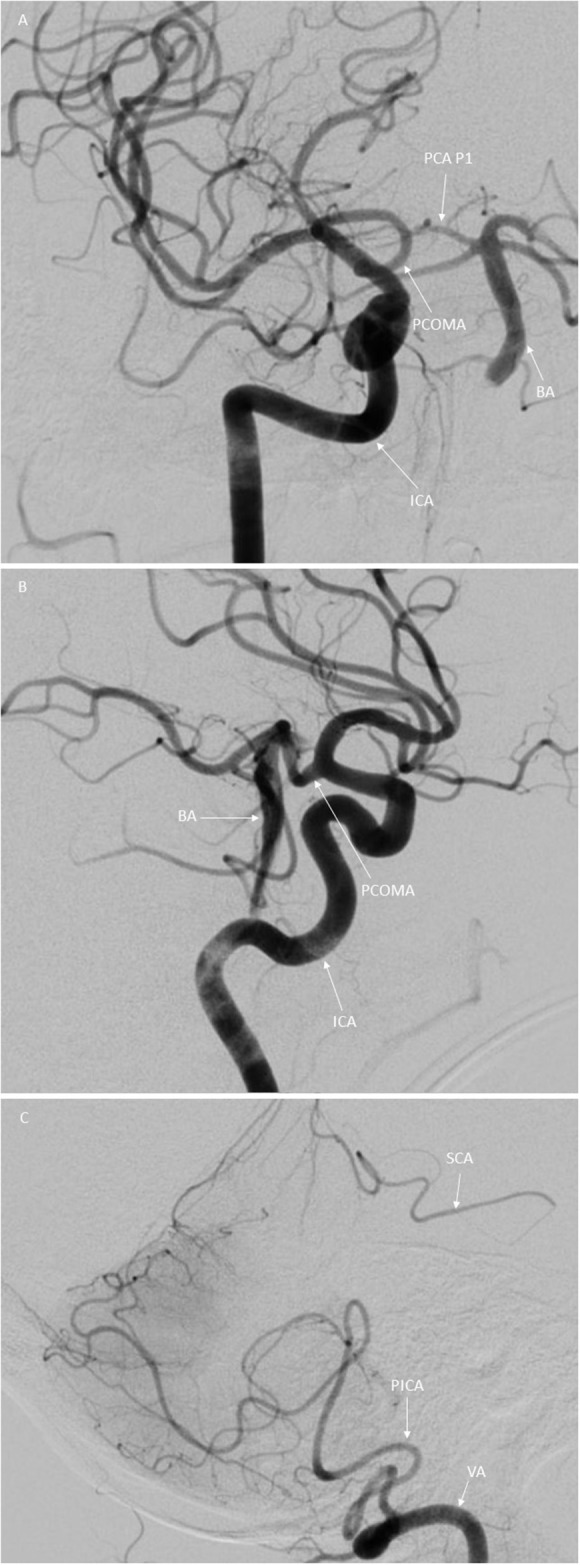
Illustration of the variants in collateral flow in aBAO in digital subtraction angiography in posterior anterior **(A)** and lateral **(B,C)** view. **(A,B)** Basilar artery (BA) with proximal occlusion indicated by the arrow. Right internal carotid artery (ICA) indicated by the arrow. Posterior communicating artery (PCOMA) indicated by the arrow. Posterior cerebral artery, first segment (PCA P1) indicated by the arrow **(C)** Right SCA-PICA collateral, right superior cerebellar artery (SCA) indicated by the arrow, right posterior inferior cerebellar artery indicated by the arrow and the right vertebral artery (VA).

The site of the arterial occlusion was defined in a binary way with either “patent” or “occluded” assigned to each artery, encompassing occlusion of the proximal, middle, distal third, or whole of the basilar artery as per standard nomenclature (13). The presence of collateral pathways was also assessed and recorded by noting unilateral or bilateral collateral flow in the aBAO via the posterior inferior cerebellar artery (PICA) and the superior cerebellar artery (SCA) pathway or the posterior communicating arteries (PCOMA) (Figure 1).

Each branch and segment of the basilar artery was reviewed as above and its patency or lack thereof noted. Both the occlusion sites and collateral status were obtained retrospectively from the angiographic DSA images by two independent reviewers (HH, JR) who were not party to the 90-day mRS. Successful recanalization was defined as TICI 2b-3. Any intraoperative or periprocedural complications were recorded.

### Post-procedural Hemorrhage

Intracranial hemorrhage (ICH) after EVT was assessed by CT and/or MRI and was rated as “asymptomatic” or “symptomatic.” “Symptomatic” was defined according to ECASS II criteria (14). Each data set was then labeled “no hemorrhage,” “asymptomatic hemorrhage,” or “symptomatic hemorrhage.” These labels were used in the statistical analysis.

All post-interventional CT or MRI images were reviewed by an independent reviewer (JR), blinded to our patient information.

### Post-procedural Follow-Up and Outcome

Post-interventional treatment management included admission to the neuro-intensive care unit or hyper acute stroke unit with routine follow-up imaging comprising either CT or MRI scans at 24–36 h and a 24 h NIHSS if possible. The mRS was assessed 24 h after EVT, at discharge, and at 90 days. The 90-day mRS was evaluated either within a follow-up clinic appointment or via telephone by trained research nurses or members of the neurological team.

### Statistical Analysis

Continuous variables were expressed as mean ± SD or median (interquartile range [IQR]) and categorical variables as numbers (percentage). Fisher's exact test was used in univariate analysis to determine whether functional outcome was independent of each variable tested. The odds ratio was outputted for each variable calculated.

**Figure 2:**
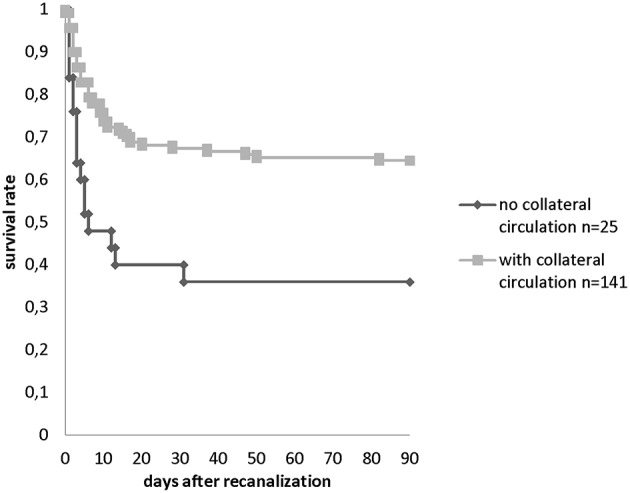
Survival rate within 90 days in patients with collaterals (*n* = 133 PCOMA and *n* = 8 SCA-PICA) and without collaterals (*n* = 25) observed in the >6 h TTR group.

Survival and functional outcome analysis for multiple variables was performed for both the ≤ 6 h time window and the >6 h time window. For the >6 h group the collateral status was drawn up (Figure 2). At this point, it shall be mentioned that this is no Kaplan Meier analysis as the follow up time is the same for every patient. The follow up interval for survival was recorded at baseline until 90 days of survival. A qualified stroke and study nurse obtained the mRS information via phone interview. Patients lost to follow up were excluded from this study.

AUC-ROC curves were calculated for admission NIHSS and 24 h NIHSS, pre-interventional PC-APECTS as per CCT or cMRI.

Wilcoxon matched pairs signed rank test was performed to test for a difference in the median NIHSS improvement (assessed at admission and 24 h after admission) stratified by mRS 0–2 and mRS 3–6.

Reliability of interobserver agreement was calculated to enable assessment of occlusion patterns and collateral status using Cohen's kappa. Value ranges from 0–0.20 as slight, 0.21–0.40 as fair, 0.41–0.60 as moderate, 0.61–0.80 as substantial, and 0.81–1 as almost perfect agreement (15).

Any data sets with discrepancies were reviewed in a group with mutual agreement achieved in each case.

A separate multivariate analysis (logistic regression) was performed for 87 patients to analyze for separate influence of early neurological improvement (assessed in NIHSS improvement) on functional outcome and mortality.

To analyze for separate influence of different covariates (gender, age, hypertension, collateral status, PCOMA status, SCA-PICA collateral status, time to recanalization, year of treatment, TICI score, and post-interventional hemorrhage) on functional outcome and mortality at 90 days logistic regression models were applied.

Level of significance was set at *p* < 0.05.

The program Stata/IC 13.1 for Windows (StataCorp LP, College Station, Texas, USA) was used for the statistical analysis.

## Results

### Background and Demographic Data

We identified 231 patients (100%) that met our inclusion criteria. Mortality at 90 days was 36.8% (*n* = 85), favorable outcome (mRS 90d 0–2) was achieved in 29.4% (*n* = 68). The median (min. – max.) age was 75 (19–110) years, with the majority of patients being male (*n* = 131, 57%). Pre-and post-interventional NIHSS at 24 h was available for 87 (100%) patients. 56 (64%) patients had a NIHSS ≥9. Out of these 56 patients 32.1% (*n* = 17) had early neurological improvement <9. The median NIHSS at admission was 14 [(0–42) *n* = 155]. Median 24 h NIHSS was 6 [(0–33) *n* = 110]. The median time to recanalization from symptom onset was 6.4 h (2.5–20.4). Only a minority of 31 patients (9.4%) received iv rtPA as the vast majority (165, 71.4%) received treatment beyond 6 h from symptom onset. In the latter group, stroke onset had not been witnessed for 81 (35.1%) patients.

### Stroke Burden/PC-ASPECTS Pre-interventional

Pre-interventional PC-ASPECTS with MRI were attained for 62 (26.8%) patients. For the majority of patients (*n* = 155, 67%) a score for pre-interventional PC-ASPECTS had to be reached via CCT.

A pre-interventional PC-ASPECTS of ≤ 7 was recorded for 24 patients based on MRI and for 13 patients using CCT. A pre-interventional ASPECTS of >7 was determined for 38 patients using MRI and for 181 patients based on CCT.

### Collaterals

Cohen's Kappa for interobserver agreement was 0.84 for the sites of occlusion and 0.91 for the collateral status.

Collateral supply was analyzed, with the dataset stratified into two groups by time to recanalization (TTR). One hundred ninety seven patients (85.3%) had collaterals. One hundred eighty three patients (79.3%) had at least one PCOMA. Thirty five patients (15.1%) were being supplied via the PICA-superior cerebellar artery (SCA) pathway. In the over 6 h group, 133 patients (57.6%) had a collateralization via at least one PCOMA while in the under 6 h TTR subgroup, 50 patients (21.6%) had at least one PCOMA.

Collateral status and pre-interventional stroke burden depicted by pre-interventional PC-ASPECTS was inconsistent regarding prediction of functional outcome and mortality. The median PC-ASPECTS with CCT was 10 irrespective of collateral status. This finding is due to the technical weakness of CCT for acute stroke in posterior fossa.

In cMRI Median PC-ASPECTS was eight if collaterals were present and 7.5 with no collaterals. This marginal effect was most likely caused by the small number of pre-interventional cMRI scans.

#### Occlusion Patterns

The most frequent occlusion pattern was the distal third 69.7% (*n* = 161) followed by occlusion of the middle third (17.3% *n* = 40) and the proximal third (3.4% *n* = 8). It was most common for one third of the basilar trunk to be occluded (45.6% *n* = 151), while occlusion of two thirds was seen in 25.1% of cases (*n* = 58). Total occlusion of the basilar trunk was recorded in 22 patients (9.5%) (Table 1).

**Table 1:** 1a Mortality over 90 days (mRS90d 0–5 vs. 6) occlusion patterns. 1b Survival over 90 days (mRS90d 0– vs. 6) and collateral status.

		***n***	**%**	**mRS 0–5**	**mRS 6**	**OR (95%-KI)**	*p*-value^*^	*p*-value^**^
**A**								
Occlusion basilar trunk	Total	22	9.5	12 (54.5%)	10 (45.5%)	Ref.	0.474	
	2/3	58	25.1	40 (69.0%)	18 (31.0%)	0.54 (0.20–1.48)		0.295
	1/3	151	65.4	100 (66.2%)	51 (33.8%)	0.61 (0.25–1.51)		0.341
Occlusion 1/3	Distal	122	80.8	79 (64.8%)	43 (35.2%)	Ref.	0.105	
	Middle	21	13.9	13 (61.9%)	8 (38.1%)	1.13 (0.43–2.95)		0.809
	Prox	8	5.3	8 (100%)	0 (0%)	-		0.052
Time to recanalization >6 h	Total	4	6.1	1 (25.0%)	3 (75.0%)	Ref.	0.036	
	2/3	24	36.4	21 (87.5%)	3 (12.5%)	0.05 (0.00–0.63)		0.022
	1/3	38	57.6	30 (78.9%)	8 (21.1%)	0.09 (0.01–0.99)		0.049
**B**								
Collateral status	No collaterals	34	14.7	15 (44.1%)	19 (55.9%)	Ref.	0.006	
	Collaterals present	197	85.3	137 (69.5%)	60 (30.5%)	0.35 (0.16–0.73)		
PCOMA	Both patent	112	49.8	85 (75.9%)	27 (24.1%)	Ref.	0.002	
	1 patent	71	31.6	42 (59.2%)	29 (40.8%)	2.17 (1.14–4.13)		0.021
	Absent	42	18.7	20 (47.6%)	22 (52.4%)	3.46 (1.64–7.30)		0.002
SCA PICA	Absent	195	84.8	129 (66.2%)	66 (33.8%)	Ref.	0.442	
	1 patent	18	7.8	13 (72.2%)	5 (27.8%)	0.75 (0.26–2.20)		0.795
	Both patent	17	7.4	9 (52.9%)	8 (47.1%)	1.74 (0.64–4.72)		0.296
PCOMA status in distal occlusion	Both patent	76	49.0	56 (73.7%)	20 (26.3%)	Ref.	0.008	
	1 patent	46	29.7	30 (65.2%)	16 (34.8%)	1.49 (0.67–3.31)		0.413
	Absent	33	21.3	14 (42.4%)	19 (57.6%)	3.80 (1.61–8.99)		0.002
PCOMA status stratified by time to recanalization >6 h	Patent	82	50.9	58 (70.7%)	24 (29.3%)	Ref.	0.003	
	Patent/absent	50	31.1	29 (58.0%)	21 (42.0%)	1.75 (0.84–3.66)		0.185
	Absent	29	18.0	10 (34.5%)	19 (65.5%)	4.59 (1.86–11.34)		0.001
Non-witnessed	Patent	40	50.6	26 (65.0%)	14 (35.0%)	Ref.	0.070	
PCOMA	Patent/absent	25	31.6	15 (60.0%)	10 (40.0%)	1.24 (0.44–3.49)		0.793
	Absent	14	17.7	4 (28.6%)	10 (71.4%)	4.64 (1.22–17.69)		0.028
Witnessed	Patent	72	49.3	59 (81.9%)	13 (18.1%)	Ref.	0.006	
PCOMA	Patent/absent	46	31.5	27 (58.7%)	19 (41.3%)	3.19 (1.38–7.42)		0.010
	Absent	28	19.2	16 (57.1%)	12 (42.9%)	3.40 (1.30–8.92)		0.019

* Global p-value,

** *p-value for each subgroup if exceeding more than two subgruops*.

### Treatment Time and Time to Recanalization

The median duration of treatment was *1.4* h (0.9–2.3). In 38.4% (*n* = 88) of cases, treatment was longer than 1 h but shorter than 2 h. In 26.2% (*n* = 60) of patients, treatment procedures lasted over 2 h. There was no effect regarding prediction of functional outcome and mortality.

Stent retrievers were used for 77.5% of patients (*n* = 179) whereas aspiration was used as the primary method of mTE in 15.2% (*n* = 35).

In 64.5% of patients, mTICI 2b-3 was achieved after 1 passage. At the end of the procedure, mTICI 2b-3 was achieved in 93.5% of patients (*n* = 221).

From 2008 to 2010, the pCRC device was predominantly used for mTE (47.2% *n* = 17), followed by Solitaire stent retrievers (25% *n* = 9), pBONNET stent retrievers (22.2% *n* = 8) and a few pREset devices (5.6% *n* = 2). In 2011 to 2013, pREset devices were used in most cases (86.5%, *n* = 90) followed by Solitaire (6.7% *n* = 7) and pBONNET devices (5.8%, *n* = 6) another 1% were other devices.

From 2014 to 02/2019, in most cases (42.8% *n* = 39) the pREset device was used followed by SOFIA Plus aspiration catheters (38.5% *n* = 35). In 14.2% of cases (*n* = 13) pREset in combination with SOFIA Plus aspiration catheters were used. In 4.3% (*n* = 4) of procedures, other stent retriever devices were used.

### Pre and Post-op Clinical and Radiological Follow-Up

Asymptomatic ICH occurred in 8.7% (*n* = 20) and symptomatic *I*CH was seen in 6% (*n* = 14) of patients.

### Statistical Analysis

**Good Outcome mRS 90 days 0–2 vs. 3–6**.

Bivariate analysis comparing good clinical outcome (mRS 0–2) with poor clinical outcome (mRS 3–6) showed no gender effect (Table 2). A positive history of smoking (*n* = 25 10.8%) was associated with a good outcome (*p* = 0.02) and diabetes mellitus (*n* = 61) was associated with poor outcome (*p* = 0.001) (Table 2). Neither a history of hypertension (*p* = 0.7) nor hypercholesterolaemia had an effect on clinical outcome (Table 2). The location of the clot in the proximal, middle, or distal third of the basilar artery did not have statistical significance for functional outcome nor did clots involving 2/3rds of the basilar artery or total occlusions (Table 3). Furthermore, there was no difference regarding different occlusion patterns of the cerebellar arteries (PICA, anterior inferior cerebellar artery AICA, SCA). NIHSS assessment at baseline could predict functional outcome. Compared to patients with 0–4 NIHSS points, patients with an NIHSS range beginning at 12 points had next to no chance of a good functional outcome. None of the patients with a baseline NIHSS (*n* = 155) of ≥28 had a good functional outcome (Table 2).

**Table 2:** Functional outcome at 90 days (mRS90d 0–2 vs. 3–6) and baseline characteristics.

		***n***	**%**	**mRS 3–6**	**mRS 0–2**	**OR (95%-KI)**	*p*-value^*^	*p*-value^**^
Gender	Female	100	43.3	67 (67.0%)	33 (33.0%)	Ref.	0.311	
	Male	131	56.7	96 (73.3%)	35 (26.7%)	0.74 (0.42–1.31)		
Age	<70	79	34.2	50 (63.3%)	29 (36.7%)	Ref.	0.155	
	70–79	84	36.4	60 (71.4%)	24 (28.6%)	0.69 (0.36–1.33)		0.316
	≥80	68	29.4	53 (77.9%)	15 (22.1%)	0.49 (0.23–1.02)		0.071
Symptom onset	Non-witnessed	82	35.5	64 (78.0%)	18 (22.0%)	Ref.	0.071	
	Witnessed	149	64.5	99 (66.4%)	50 (33.6%)	1.80 (0.96–3.36)		
Diabetes mellitus	No	128	67.7	79 (61.7%)	49 (38.3%)	Ref.	0.001	
	Yes	61	32.3	52 (85.2%)	9 (14.8%)	0.28 (0.13–0.62)		
Cholesterol	No	130	68.8	94 (72.3%)	36 (27.7%)	Ref.	0.130	
	Yes	59	31.2	36 (61.0%)	23 (39.0%)	1.67 (0.87–3.20)		
Smoker	No	159	86.4	115 (72.3%)	44 (27.7%)	Ref.	0.020	
	Yes	25	13.6	12 (48.0%)	13 (52.0%)	2.83 (1.20–6.69)		
Hypertension	No	38	17.4	28 (73.7%)	10 (26.3%)	Ref.	0.844	
	Yes	180	82.6	127 (70.6%)	53 (29.4%)	1.17 (0.53–2.58)		
Pre-PCASPECT cMRI	≤ 7	24	38.7	18 (75.0%)	6 (25.0%)	Ref.	0.188	
	>7	38	61.3	22 (57.9%)	16 (42.1%)	2.18 (0.70–6.79)		
Pre-PCASPECT cCT	≤ 7	13	8.4	10 (76.9%)	3 (23.1%)	Ref.	0.760	
	>7	142	91.6	101 (71.1%)	41 (28.9%)	1.35 (0.35–5.19)		
Admission NIHSS	0–4	27	17.4	7 (25.9%)	20 (74.1%)	Ref.	<0.001	0.067
	5–11	35	22.6	18 (51.4%)	17 (48.6%)	0.33 (0.11–0.98)		<0.001
	12–22	53	34.2	44 (83.0%)	9 (17.0%)	0.07 (0.02–0.22)		<0.001
	23–27	18	11.6	16 (88.9%)	2 (11.1%)	0.04 (0.01–0.24)		<0.001
	≥28	22	14.2	22 (100%)	0 (0%)			

* Global p-value,

** *p-value for each subgroup if exceeding more than two subgruops*.

**Table 3:** Functional outcome at 90 days (mRS90d 0–2 vs. 3–6), occlusion patterns, and collateral status.

		***n***	**%**	**mRS 3–6**	**mRS 0–2**	*p*-value^*^	**OR (95%-KI)**	*p*-value^**^
Occlusion basilar trunk	Total	22	9.5	15 (68.2%)	7 (31.8%)	0.791	Ref.	
	2/3	58	25.1	43 (74.1%)	15 (25.9%)		0.75 (0.26–2.19)	0.588
	1/3	151	65.4	105 (69.5%)	46 (30.5%)		0.94 (0.36–2.46)	1.000
Occlusion 1/3	Distal	122	80.8	87 (71.3%)	35 (28.7%)	0.571	Ref.	
	Middle	21	13.9	13 (6–1.9%)	8 (38.1%)		1.53 (0.58–4.02)	0.442
	Proximal	8	5.3	5 (62.5%)	3 (37.5%)		1.49 (0.34–6.61)	0.691
		***n***	**%**	**mRS 3–6**	**mRS 0–2**	**OR (95%-KI)**	***p***-value^*^	***p***-value^**^
Collateral status	No collaterals	34	14.7	29 (85.3%)	5 (14.7%)	Ref.	0.043	
	Collaterals	197	85.3	134 (68.0%)	63 (32.0%)	2.73 (1.01–7.39)		
PCOMA	Patent	112	49.8	70 (62.5%)	42 (37.5%)	Ref.	0.049	
	Patent/absent	71	31.6	53 (74.6%)	18 (25.4%)	0.57 (0.29–1.09)		0.107
	Absent	42	18.7	34 (81.0%)	8 (19.0%)	0.39 (0.17–0.93)		0.034
SCA PICA	Absent	195	84.8	139 (71.3%)	56 (28.7%)	Ref.	0.757	
	1 patent	18	7.8	12 (66.7%)	6 (33.3%)	1.24 (0.44–3.48)		0.787
	Both patent	17	7.4	11 (64.7%)	6 (35.3%)	1.35 (0.48–3.85)		0.584
PCOMA status in distal occlusion	Both patent	76	49.0	47 (61.8%)	29 (38.2%)	Ref.	0.034	
	1 patent	46	29.7	35 (76.1%)	11 (23.9%)	0.51 (0.22–1.16)		0.116
	Absent	33	21.3	28 (84.8%)	5 (15.2%)	0.29 (0.10–0.84)		0.024
PCOMA status in proximal and middle occlusion	Both patent	29	60.4	19 (65.5%)	10 (34.5%)	Ref.	0.397	
	1 patent	18	37.5	13 (72.2%)	5 (27.8%)	0.73 (0.20–2.68)		0.753
	Absent	1	2.1	0 (0%)	1 (100%)			0.367
Non-witnessed onset and	Patent	40	50.6	27 (67.5%)	13 (32.5%)	Ref.	0.130	
PCOMA	Patent/absent	25	31.6	22 (88.0%)	3 (12.0%)	0.28 (0.07–1.13)		0.080
	Absent	14	17.7	12 (85.7%)	2 (14.3%)	0.35 (0.07–1.80)		0.302
Witnessed onset and	Patent	72	49.3	43 (59.7%)	29 (40.3%)	Ref.	0.205	
PCOMA	Patent/absent	46	31.5	31 (67.4%)	15 (32.6%)	0.72 (0.33–1.56)		0.440
	Absent	28	19.2	22 (78.6%)	6 (21.4%)	0.40 (0.15–1.12)		0.103
Time to recanalization ≤ 6 h	Patent	30	46.9	15 (50.0%)	15 (50.0%)	Ref.	0.483	
	Patent/absent	21	32.8	14 (66.7%)	7 (33.3%)	0.50 (0.16–1.60)		0.266
	Absent	13	20.3	8 (61.5%)	5 (38.5%)	0.63 (0.16–2.38)		0.526

* Global p-value,

** *p-value for each subgroup if exceeding more than two subgruops*.

This finding was more marked in NIHSS assessment at 24 h (*n* = 110), as none of these patients assessed with NIHSS ≥ 23 achieved a good functional outcome (Table 4).

**Table 4:** Functional outcome at 90 days (mRS90d 0–2 vs. 3–6) and post-interventional outcome screening.

		***n***	**%**	**mRS 3–6**	**mRS 0–2**	**OR (95%-KI)**	*p*-value^*^	*p*-value^**^
NIHSS at 24 h	0–4	42	38.2	12 (28.6%)	30 (71.4%)	Ref.	<0.001	
	5–11	20	18.2	10 (50.0%)	10 (50.0%)	0.40 (0.13–1.21)		0.155
	12–22	28	25.5	26 (92.9%)	2 (7.1%)	0.03 (0.01–0.15)		<0.001
	23–27	17	15.5	17 (100%)	0 (0%)			<0.001
	≥28	3	2.7	3 (100%)	0 (0%)			0.032
Post-interventional ICH	No	193	85.0	131 (67.9%)	62 (32.1%)	Ref.		0.014
	Yes	34	15.0	30 (88.2%)	4 (11.8%)	0.28 (0.09–0.84)		
Post-interventional sICH	No	190	93.1	136 (71.6%)	54 (28.4%)			0.023
	Yes	14	6.9	14 (100%)	0 (0%)			

* Global p-value,

** *p-value for each subgroup if exceeding more than two subgruops*.

A trend was observed for pre-interventional PC-ASPECTS. Patients with a cMRI PC-ASPECTS of >7 had a twofold chance of a good functional outcome. This effect was not significant due to the small sample size in MRI. Pre-interventional PC-ASPECTS using CT scans were inconclusive (Table 2).

AUC-ROC analysis of duration of treatment as a continuous variable did not differ in terms of predictive value regarding functional outcome. Neither did pre-interventional PC-ASPECT with CT or MRI.

Out of 87 patients with baseline and 24 h NIHSS 31 patients (35.6%) experienced good outcome and 56 patients (64.4%) had an unfavorable outcome. The median 24 h NIHSS for the patient group with favorable outcome was two compared to 20 in the group with unfavorable outcome. The difference in median of pre-interventional and 24 h NIHSS was significant (*p* < 0.001).

NIHSS at admission and at 24 h had a very good and significant discriminative power regarding good functional outcome in AUC-ROC analysis (ROC *p* < 0.001). AUC for each was >0.8.

Patients with TTR beyond 6 h had less than half the chance of good outcome (*p* = 0.025) compared to patients with a TTR of <6 h (Table 5). Patients with collaterals had an almost triple likelihood of a good outcome (*p* = 0.043) (Table 3). The probability of a good functional outcome was reduced by more than half if PCOMA was absent (Table 3). There was also a slight, non-significant correlation of a good outcome and PICA-SCA collaterals being present, whether these were unilateral or bilateral (Table 3).

**Table 5:** Functional outcome at 90 days (mRS90d 0–2 vs. 3–6) and procedural parameters.

		***n***	**%**	**mRS 0–2**	**mRS 3–6**	**OR (95%-KI)**	*p*-value^*^	*p*-value^**^
Time to recanalization [h]	≤ 6 h	66	28.6	39 (59.1%)	27 (40.9%)	Ref.	0.025	
	>6 h	165	71.4	124 (75.2%)	41 (24.8%)	0.48 (0.26–0.88)		
Witnessed	Non-witnessed	82	35.5	64 (78.0%)	18 (22.0%)	Ref.	0.071	
	Witnessed	149	64.5	99 (66.4%)	50 (33.6%)	1.80 (0.96–3.36)		
≤ 6 h	Witnessed	65	100.0	38 (58.5%)	27 (41.5%)			
>6 h	Non-witnessed	82	49.4	64 (78.0%)	18 (22.0%)	Ref.	0.474	
	Witnessed	84	50.6	61 (72.6%)	23 (27.4%)	1.34 (0.66–2.73)		
Duration of	<1 h	81	35.4	53 (65.4%)	28 (34.6%)	Ref.	0.256	
Treat. [h]	≥1 h	88	38.4	62 (70.5%)	26 (29.5%)	0.79 (0.41–1.52)		0.512
	≥2	60	26.2	47 (78.3%)	13 (21.7%)	0.52 (0.24–1.13)		0.133
Year of treatment	2008–2010	36	15.6	32 (88.9%)	4 (11.1%)	Ref.	0.009	
	2011–2019	195	84.4	131 (67.2%)	64 (32.8%)	3.91 (1.32–11.55)		

* Global p-value,

** *p-value for each subgroup if exceeding more than two subgruops*.

Surprisingly, the odds of a good functional outcome were reduced by 70% if PCOMA was absent through a distal occlusion in the basilar artery. However, the proximal or middle third being occluded did not have any effect on good functional outcome, this finding is most likely due to a small sample size (Table 3).

Post-interventional hemorrhage decreased the likelihood of good functional outcome by more than 70% This latter effect was particularly pronounced in patients with symptomatic post-interventional intracranial hemorrhages (sICH) as 100% did not achieve good functional outcomes (Table 4).

Multivariate analysis revealed both the time to recanalization and ICH to be predictors for functional outcome at 90 days. The likelihood of a good outcome was reduced by half if TTR was >6 h and by more than 70% if post-interventional hemorrhage was found (Table 6).

**Table 6:** Multivariate analysis mRS 0–2 vs. 3–6 (*n* = 208).

		**OR (95%-KI)**	*p-*value^*^
Gender	Male vs. female	0.85 (0.44–1.64)	0.634
Age	70–79 vs <70	0.64 (0.29–1.41)	0.268
	≥80 vs. <70	0.56 (0.24–1.29)	0.173
Hypertension	Yes vs. no	1.17 (0.51–2.73)	0.709
Collaterals	Present vs. not present	1.01 (0.17–5.92)	0.995
Time to recanalization	>6 h vs. ≤ 6 h	0.47 (0.23–0.95)	0.036
Year of treatment	From 2011 vs. 2008–2010	3.95 (1.14–13.66)	0.030
Post image hemorrhage	Yes vs. No	0.28 (0.08–0.98)	0.046
PCOMA status	Unilateral vs. bilateral	0.54 (0.27–1.11)	0.094
	Non vs. bilateral	0.43 (0.09–1.97)	0.275

* *Global p-value*.

Patients treated from 2011 to the beginning of 2019 had an almost fourfold likelihood of a good outcome in comparison to patients treated in the earlier period of 2008–2010 (Table 6).

In a separate multivariate analysis with 87 patients NIHSS improvement was an independent predictor for good functional outcome [OR 1.28 (1.14–1.45) *p* < 0.001]. Latter finding should be interpreted with caution as the NIHSS was more likely missing for patients with mRS 90d 3–6.

### Mortality

In bivariate analysis Risk of death over 90 days was high if the NIHSS had a value of 12–22 points, both for NIHSS at admission and at 24 h after admission. In each case, there was a 6-fold risk compared to patients with an NIHSS of 0–4 (Table 7).

**Table 7:** Mortality over 90 days (mRS90d 0–5 vs. 6) and baseline assessment.

		***n***	**%**	**mRS 0–5**	**mRS 6**	**OR (95%-KI)**	*p*-value^*^	*p*-value^**^
Gender	Female	100	43.3	70 (70.0%)	30 (30.0%)	Ref.	0.265	
	Male	131	56.7	82 (62.6%)	49 (37.4%)	1.39 (0.80–2.43)		
Alter	<70	79	34.2	56 (70.9%)	23 (29.1%)	Ref.	0.467	
	70–79	84	36.4	52 (61.9%)	32 (38.1%)	1.50 (0.78–2.89)		0.249
	≥80	68	29.4	44 (64.7%)	24 (35.3%)	1.33 (0.66–2.66)		0.480
Pre-interventional NIHSS	0–4	27	17.4	24 (88.9%)	3 (11.1%)	Ref.	0.005	
	5–11	35	22.6	28 (80.0%)	7 (20.0%)	2.00 (0.46–8.64)		0.491
	12–22	53	34.2	29 (54.7%)	24 (45.3%)	6.62 (1.77–24.80)		0.002
	23–27	18	11.6	11 (61.1%)	7 (38.9%)	5.09 (1.10–23.61)		0.064
	≥28	22	14.2	12 (54.5%)	10 (45.5%)	6.67 (1.53–28.97)		0.010
Pre-interventional Aspect MRI	≤ 7	24	38.7	13 (54.2%)	11 (45.8%)	Ref.	0.051	
	>7	38	61.3	30 (78.9%)	8 (21.1%)	0.32 (0.10–0.97)		
Pre-interventional Aspect CT	≤ 7	13	8.4	9 (69.2%)	4 (30.8%)	Ref.	1.000	
	>7	142	91.6	93 (65.5%)	49 (34.5%)	1.19 (0.35–4.06)		

* Global p-value,

** *p-value for each subgroup if exceeding more than two subgruops*.

Adjusted for 90-day mortality, the odds of death were reduced by more than half if collaterals were present. Collateral status did matter in the TTR over 6 h subgroup. The odds were reduced by 65 percent when compared to patients with no collaterals (Table 1).

If PCOMA was absent, the likelihood of death was 3.4 times as high as for patients with bilateral PCOMA present (Table 1). Patients with unilateral PCOMA had double the risk of dying compared to patients with bilateral PCOMA (Table 1). Stratified by occlusion patterns, patients with an occluded distal third had almost a four times higher risk of death if their PCOMA were also absent compared to patients who still had recourse to bilateral PCOMA (Table 1). Also considering the time window to treatment, the patients in the >6 h TTR subgroup whose PCOMA were also absent had a 4.5 times higher likelihood of dying compared to members of the same subgroup who still had both PCOMA present (Table 1). Furthermore, in bivariate analysis, the PCOMA status showed a trend toward helping predict mortality in strokes with both unwitnessed and witnessed onsets. This effect was significant for witnessed onsets and almost for unwitnessed. The likelihood of death was more than threefold higher if PCOMA was absent (Table 1).

Regarding occlusion patterns observed in the ≤ 6 h TTR the likelihood for death was reduced to more than 90% in comparison to total occlusion if partial occlusion was noted. This effect was not observed in the >6 h TTR (Table 1).

For patients in the TTR >6 h subgroup, we observed that likelihood of death was increased by a factor of 2.4 compared to the TTR within 6 h group (Table 8).

**Table 8:** Mortality over 90 days (mRS90d 0–5 vs. 6) and periprocedural parameters.

		***n***	**%**	**mRS 0–5**	**mRS 6**	**OR (95%-KI)**	*p*-value^*^	*p*-value^**^
TTR [h]	≤ 6 h	65	28.1	52 (80.0%)	13 (20.0%)	Ref.	0.005	
	>6 h-m	166	71.9	100 (60.2%)	66 (39.8%)	2.64 (1.33–5.23)		
pCRC device	No	214	92.6	146 (68.2%)	68 (31.8%)	Ref.	0.014	
	yes	17	7.4	6 (35.3%)	11 (64.7%)	3.94 (1.39–11.11)		
TICI score	0–2a	12	5.2	3 (25.0%)	9 (75.0%)	Ref.	0.004	
	2b-3	219	94.8	149 (68.0%)	70 (32.0%)	0.16 (0.04–0.60)		
Time to recanalization >6 h	0–2a	11	6.7	2 (18.2%)	9 (81.8%)	Ref.	0.007	
	2b-3	154	93.3	98 (63.6%)	56 (36.4%)	0.13 (0.03–0.61)		
Year of treatment	2008–2010	36	15.6	16 (44.4%)	20 (55.6%)	Ref.	0.007	
	2011–2019	195	84.4	136 (69.7%)	59 (30.3%)	0.35 (0.17–0.72)		

* Global p-value,

** *p-value for each subgroup if exceeding more than two subgruops*.

Looking at TICI scores, patients with a TICI of 2b-3 had a 80% lower probability of dying in comparison to patients with a TICI of 0–2a (Table 8).

If post-interventional hemorrhage occurred, these patients had an over 4-fold probability of dying. This effect was even more pronounced in patients with symptomatic hemorrhage (Table 9).

**Table 9:** Survival over 90 days (mRS90d 0–5 vs. 6) and post-interventional outcome.

		***n***	**%**	**mRS 0–5**	**mRS 6**	*p*-value^*^	**OR (95%-KI)**	*p*-value^**^
NIHSS at 24 h	0–4	42	38.2	38 (90.5%)	4 (9.5%)	Ref.	<0.001	
	5–11	20	18.2	19 (95.0%)	1 (5.0%)	0.50 (0.05–4.84)		1.000
	12–22	28	25.5	17 (60.7%)	11 (39.3%)	6.15 (1.70–22.22)		0.006
	23–27	17	15.5	4 (23.5%)	13 (76.5%)	30.87 (6.69–142.48)		<0.001
	≥28	3	2.7	2 (66.7%)	1 (33.3%)	4.75 (0.34–65.52)		0.304
Post – interventional ICH	No	193	85.0	136 (70.5%)	57 (29.5%)	Ref.	<0.001	
	Yes	34	15.0	12 (35.3%)	22 (64.7%)	4.37 (2.03–9.45)		
Post – interventional sICH	No	190	93.1	128 (67.4%)	62 (32.6%)	Ref.	<0.001	
	Yes	14	6.9	3 (21.4%)	11 (78.6%)	7.57 (2.03–28.21)		

* Global p-value,

** *p-value for each subgroup if exceeding more than two subgruops*.

Furthermore patients who were treated from the beginning of 2011 had mortality reduced to 0.35 (0.14–0.65) (*p* < 0.009) compared to patients treated in the years 2008–2010 (Table 8).

In multivariate analysis, independent influencing factors for mortality were post-interventional ICH (*p* < 0.001), collateral status (*p* = 0.03), and TICI score (*p* = 0.02) (Table 10). In a separate multivariate analysis with 87 patients NIHSS improvement was an independent predictor for mortality [OR 0.84 (0.77–0.92) *p* < 0.001]. Latter finding should be interpreted with caution as the NIHSS was more likely missing for patients with mRS 90d 3–6.

**Table 10:** Multivariate analysis mortality (*n* = 208).

		**OR (95%-KI)**	*p*-value^*^
Gender	Male vs. female	1.46 (0.74–2.9)	0.277
Age	70–79 vs. <70	1.68 (0.75–3.79)	0.208
	≥80 vs. <70	1.29 (0.52–3.2)	0.589
Hypertension	Yes vs. no	0.78 (0.32–1.91)	0.582
Collaterals	Present vs. not present	0.16 (0.03–0.87)	0.034
Time to recanalization	>6 h vs. ≤ 6 h	1.98 (0.8–4.93)	0.141
Year of treatment	From 2011 vs. 2008–2010	0.57 (0.21–1.58)	0.280
Post-image hemorrhage	Yes vs. No	5.35 (2.20–12.98)	<0.001
PCOMA status	Unilateral vs. bilateral	2.89(1.37–6.09)	0.005
	Non vs. bilateral	0.99 (0.22–4.53)	0.994
TICI	2b-3 vs. 0–2a	0.19 (0.05–0.78)	0.021

* *Global p-value*.

## Discussion

In our study on aBAO certain trends correlating to good functional outcome and to mortality over 90 days were observed.

In this respect, the 6 h TTR and post-interventional hemorrhage were most predictive for functional outcome at 90 days. In bivariate analysis, collateral status, diabetes mellitus, smoking status, year of treatment, admission NIHSS and 24 h NIHSS could predict functional outcome.

As regards mortality, in multivariate analysis the strongest predictive power was found for collateral/PCOMA status, post-interventional hemorrhage, and TICI score. In bivariate analysis, the following variables were associated with mortality over 90 days: collateral status (in particular collateral status in the beyond 6 h TTR group), devices used, year of treatment, sICH, admission NIHSS and 24 h NIHSS. The observed mortality rate was in line with other reports (1, 2).

The success of multiple recent trials (16–22) has led to mechanical thrombectomy becoming the gold standard of treatment for proximal large vessel occlusion of the anterior circulation. There are currently no randomized controlled trials to suggest that mechanical thrombectomy is a superior standard treatment for occlusions of the posterior circulation. Many centers perform mechanical thrombectomy for acute occlusions of the posterior circulation. However, the optimal selection criteria are yet to be identified. The current American Heart Association guidelines define them as a groin puncture time within 6 h of symptom onset with causative occlusion of the basilar artery (class IIb; level of evidence C) (23). There is conflicting evidence on the importance of the timing in aBAO. Several previous reports have suggested recanalization ≤ 6 h (24, 25) improves outcome whilst others have suggested the 6 h time window has little effect (2, 26, 27). The largest multicenter study with 592 patients in aBAO reported the worst outcome if recanalization took over 9 h (3). The same research group found that their patients had the best outcome if time to recanalization was <6 h (24). Recently published data (28) with 376 patients from the same registry found that time to treatment of more than 6 h was predictive of poor outcome. A multicenter report (27) with 100 patients found that time to treatment of <6 h was predictive of favorable outcome in patients with TICI 2b-3 after intervention.

Antithetically, there are findings from a large single center report with 184 patients by Strbian et al. (26) that suggest time to recanalization does not predict outcome. There was no information on the extent of the occlusion in aBAO or on collateral status and only a minority of patients−7%—received endovascular treatment. The Endostroke multi-center study (2) encompassing 148 patients could not predict outcome through time to recanalization. Information on the collateral status of individuals treated beyond 6 h was not collected. A recently published study (27) with 215 patients from two endovascular centers also could not predict outcome based on time of onset to treatment. Collateral status and type of occlusion were not factors considered.

In our study, patients with recanalization within <6 h from symptom had the best outcome. This finding is in line with the BASICS registry.

Now the reasonable question for the ambiguous findings regarding the TTR arises. Subsequently potential causes shall be discussed.

### Collateral Status

It is widely accepted that pial collaterals are important in preserving brain tissue in anterior circulation strokes. However, published literature for aBAO in this context is scarce.

A study (29) with 149 patients found that PCOMA status could predict outcome at 1 month. Furthermore, there are few grading systems under discussion that link PCOMA status and clot burden to functional outcome (30, 31). None of these studies link their findings to the TTR.

In our study, we could observe the pronounced impact of collateral status on functional outcome and mortality, in particular in the >6 h TTR group. This might be due to the fact that collaterals become more vital the longer a vessel occlusion is present.

In terms of occlusion patterns it is a surprising finding that the presence of collaterals when the distal third is occluded is associated with both a good functional outcome and with reduced mortality. This might be pathophysiologically explained by head loss in the basilar artery and the subsequent suction effect from the anterior circulation via PCOMA. The missing effect of collaterals on other occlusion patterns is most likely a sample size issue. Moreover, distal occlusions were most frequently seen.

Another controversial debate in aBAO concerns thrombus length, functional outcome and survival. In a small study (32) with 32 patients, thrombus length was an independent marker for an unfavorable outcome. Other studies investigating length were not in line with this finding (33–35), however, two of these studies were explicitly looking at monosegmental occlusion of the basilar trunk. The one study (35) considering occlusion of two segments or more had a small sample size of 40 patients and found a trend of improved outcome or survival related to the extent of the occlusion in aBAO.

In our study, the extent to which the basilar artery was occluded did not predict functional outcome but could predict mortality if applied to the ≤ 6 h time window to treatment. Latter finding should be interpreted with caution as only three patients had a total occlusion.

In our study, successful reperfusion conveyed through a TICI score was a separate predictor for mortality. This finding, that patients with successful recanalization had a lower mortality rate, is in line with current literature (27). A limitation of our finding is the small sample size in the unsuccessful reperfusion group.

### Pre-and Post-interventional Assessments

Rangaraju et al. (28) found that NIHSS at 24–48 h could predict functional outcome for 1 month in aBAO. Our study confirms this finding, extends the validity of this finding to 90 days and adds admission NIHSS as a further powerful tool to predict functional outcome over 90 days.

The results regarding early neurologic improvement were inconclusive due to a small sample size. Nevertheless a trend toward NIHSS improvement and functional outcome as well as mortality was observed.

In current literature, pre ASPECT scores for the posterior circulation are the subject of discussion. All share the view that native CCT is not suitable for evaluation of the PC-ASPECT. As in our study the vast majority received pre-interventional CCT pre-interventional PC-ASPECT scores were not predictive of functional outcome. Nevertheless, in the small number of patients who has a pre-interventional cMRI, a trend for predicting functional outcome using the PC-ASPECT score could indeed be seen. This latter trend is in line with current findings in literature (12, 36).

As recently published, symptomatic intracranial hemorrhage is associated with an unfavorable outcome (37, 38). In our study, intracranial hemorrhage was highly predictive for both an unfavorable functional outcome and for mortality. This might be due to the high percentage (41%) of symptomatic hemorrhages among ICH cases.

Furthermore, the year of treatment showed an impact on both functional outcome and mortality in bivariate analysis. This can be ascribed to the old fashioned pCRC devices still pre-dominantly used in mechanical thrombectomies in the period 2008–2010.

Furthermore, various patient characteristics are currently being discussed as possible predictors of outcome. A positive smoking status is being considered as a predictor of good outcome and is also known as the smoking paradox (27, 39). In our study, only 25 patients were smokers and generally relatively young, which might explain the better outcome (38, 40). Furthermore, this correlation disappeared in multivariate analysis.

In our cohort, diabetes mellitus was a predictor for poor functional outcome in bivariate analysis. Most reports (7, 25, 27, 28, 39, 41) on this topic are not in line with this observation in aBAO, instead suggesting that HbA1c could be a better parameter for comparison. Our data did not include information on HbA1c.

Limitations of this study are the retrospective single center design. A strength of this study is its sample size, to date the highest to be published from a single center.

## Conclusion

Our data suggest that the time window to treatment, collateral status, reperfusion status, extent of occlusion and ICH in aBAO can predict functional outcome and mortality.

The most important finding of this study is that the time window to successful treatment seems to be dependent on collateral status and extent of occlusion. These findings may serve as an explanation for the ambiguous findings regarding the validity of the 6 h time window in aBAO. They might also be helpful for decision making regarding EVT in aBAO, especially as an increasing number of patients are being referred from primary or secondary care hospitals to specialized neuro centers where time from symptom onset is often considered when deciding how to best treat an aBAO. Furthermore, baseline NIHSS, NIHSS at 24 h and post-interventional ICH were good predictors for mRS at 90 days.

## Data Availability

The datasets generated for this study are available on request to the corresponding author.

## Ethics Statement

The study was approved by the ethics committee of the Regional Medical Board Baden-Württemberg, project number F-2012-077.

Patient consent was not obtained as data were analyzed anonymously.

## Author Contributions

JR and HH contributed to the conception and design of the work, literature search, analysis and interpretation, and article drafting. MA, VH, PB, HB, and HH contributed to critical revision. All authors gave final approval of the version to be published.

### Conflict of Interest Statement

MA and PB have proctoring and consulting agreements with phenox GmbH. HH is co-founder and shareholder of phenox GmbH and femtos GmbH. JR, VH, and HB report no potential conflict of interest.
